# Updimensioning strategy derived from synthetic equiaxed grain structures for approximating 3D grain size distributions from 2D visualizations with 1D parameters

**DOI:** 10.1038/s41598-024-73090-8

**Published:** 2024-10-03

**Authors:** Kevin Gillespie, Algirdas Baskys, Ian Pong, Jean-Francois Croteau

**Affiliations:** 1 Superconducting Magnets Group, Lawrence Berkeley National Laboratory, Berkeley, CA 94720 USA; 2 European Organization for Nuclear Research, Genève 23, 1211 Geneva, Switzerland

**Keywords:** Materials science, Techniques and instrumentation, Theory and computation

## Abstract

We generated synthetic equiaxed grain structures using computer graphics software to explore the relationship between various grain size determination methods and true three-dimensional (3D) grain diameters. Mirroring grain measurement techniques, the synthetic 3D grain structures are imaged as 2D micrographs which are measured to yield 1D grain size parameters. Synthetic grain structures provide data at a mass scale and permit exploration of both polished and fractured surface micrographs, revealing one-to-one correspondence between exposed 2D grain cross-sections and individual 3D grains. Analysis of this correspondence yielded a procedure to approximate 3D equiaxed grain size and volume distributions based on the mode of the 2D fractograph grain size distribution. The 3D approximation procedure is shown to be less susceptible to different imaging conditions that affect small, undiscernible grains compared to the standard planimetric and linear intercept methods, which by design also tend to underestimate the 3D grain diameter. The procedure requires larger sample sizes to lower variance and a deeper analysis which could become more practical with machine learning (ML) models for grain boundary segmentation, which synthetic grain structures can help train. This work lays the foundation for analyzing other grain distributions such as columnar and composite grains in similar depth.

## Introduction

No high-volume engineering material is “perfect” and completely defect free in its crystal structure. Grain boundaries, a type of lattice imperfection or defect, separate grains: regions of material with a shared crystallographic orientation. Processes introducing engineered defects such as grain boundaries into the lattice of a material are often desirable to achieve specific properties. For example, small grains lead to a higher yield stress, following the so-called Hall-Petch effect which is well applied in structural material analysis^1,2^. The generation and usage of synthetic grain structures permits a more detailed and easily scalable study of grain microstructure by connecting the 3D “ground truth” to the grain size measurement methods employed on 2D cross-sections. The current study leads us to propose an alternative method for measuring and approximating equiaxed grain size and volume distributions which could prove less susceptible to different experimental conditions and unpredictable imaging noise.

Grain size can be determined by a range of techniques and methods, from those that have been practiced by metallurgists for millennia to those that were invented in recent decades. Most studies of grain size are conducted stereologically by imaging a material surface with a microscope, which is when aberration, noise, surface unevenness, and other factors can introduce artifacts. The surface is often sectioned and polished (and chemically etched when appropriate). When a material is brittle and intergranular fracture is predominant, a surface may also be fractured to yield a fractograph. After the target features (e.g. grain boundaries) are identified and segmented, the American Society for Testing and Materials (ASTM) standard methods such as the Heyn line-intercept and Saltykov planimetric methods are commonly performed on these cross-sections to determine the grain size as a 1D diameter-equivalent parameter^3^. Some of these methods can yield grain size distributions, though average values are often sufficient and reported^4^.

Thus, grain size is routinely determined by viewing and measuring an ensemble of discernible, lower dimensional grain area projections. This dimension reduction of the problem comes with a loss of information that is often necessary due to the difficulty and high expense of obtaining physical 3D grain information^3^. The further reduction of 2D cross-sections to a 1D diameter-equivalent parameter can be advantageous to establish relationships between grain features and materials properties. For example, the detection of fine-grain emergence during high temperature superplastic forming was sufficient evidence of dynamic recrystallization^5,6^.

However, for some applications and for advancing materials science, the insufficiency arising from the loss of information will become evident. Complications arise in applications where the grain sizes examined within and across different studies require better precision. Smaller grains and larger spread both approach a limit where microscope performance and image acquisition artifacts become a non-negligible factor in grain size determination, thus affecting the comparisons. For example, in Nb_3_Sn superconductors, very small grain sizes increase the maximum supercurrent carrying capability by a phenomenon called “flux-pinning”^7^. Differing determination methods and uncertain artifacts during image analysis would complicate the quantitative understanding of the effects of novel design parameters on the Nb_3_Sn wire performance. Analysis of synthetic grain structures suggests an alternative procedure for determining the true 3D grain size parameter that is more robust to these effects.

### Synthetic modeling of grain structures

According to many experimental studies, grain volume distributions for equiaxed, single phase crystallographic materials are unimodal and right skewed, modeled by log-normal or gamma distributions^8–10^. Log-normal distributions model multiplicative effects such as volume in grain-growth kinetics, where larger volumes grow faster due to an expanding surface area. The gamma distribution description of the grain volume density function stems from the cell-growth model. If grain volumes are converted into a 1D equivalent diameter, the resulting distribution is less skewed. When surveying the literature, it is important to maintain a distinction between 1D, 2D, and 3D parameters and whether they are lower-dimensional projections (e.g. cords, cross-section areas) or equivalent diameters, as these details influence the resulting density function for the parameter.

Synthetic modeling of grain structures ranges from packing standard shapes, to Voronoi tessellation techniques, to more expensive but realistic grain-growth kinetics models such as Montecarlo-Potts^11^. Voronoi tessellation involves the sampling of random nucleation sites and creating individual grains out of the regions of space closest to each of these sites. The widely accepted Voronoi tessellation methods for modeling grains can use a Euclidian distance metric (Poisson, Hardcore) or a more configurable power metric (Laguerre) which can account for more grain irregularity and larger grains^12^. We use a Poisson Voronoi tessellation^13^ to provide lower structural variance compared to other techniques, which allows us to draw broader conclusions about the fundamental properties of stereological methods.

The Poisson Voronoi technique creates a roughly symmetric distribution of 1D spherical equivalent diameters^14^, which corresponds with a distribution of 3D grain volumes that is approximately gamma^15^. Poisson Voronoi generation has been used to model material deformation^14^ and calculate physical constants such as thermal expansion coefficients^16^. The findings from this synthetic grain model could be leveraged to enhance more advanced Laguerre or Hardcore Voronoi models that use optimization procedures to capture the properties of specific materials.

**Fig. 1:**
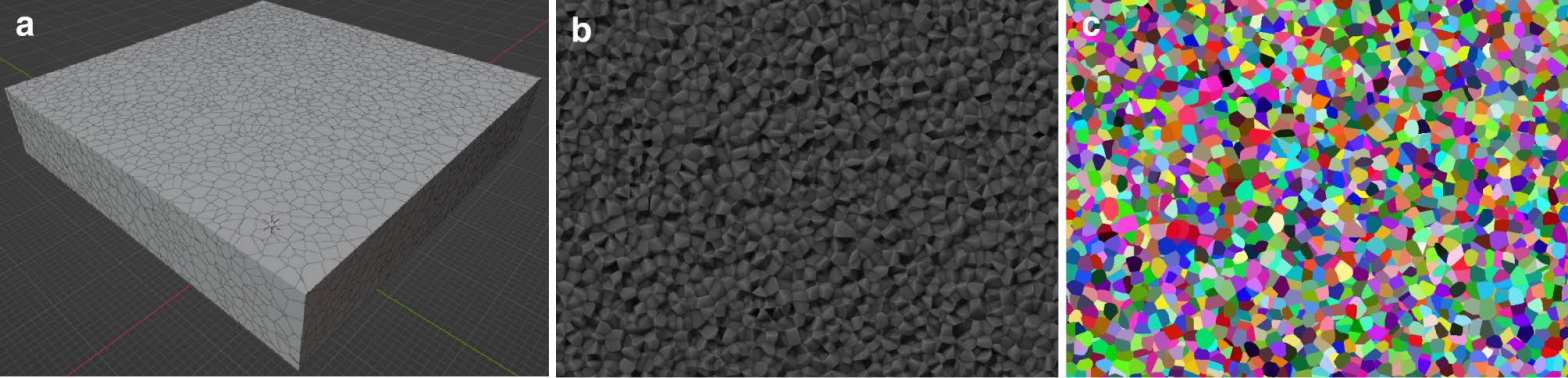
Three steps of the synthetic structure generation pipeline. (**a**) A synthetic grain structure in the 3D environment. (**b**) Fractograph image showing the geometry of a synthetic cross-section. (**c**) A colorized cross-section for image analysis. Each grain has a distinct color for unique identification and one-to-one correspondence with its 3D counterpart.

The synthetic grain structures (Fig. 1) allow for rapid simulation and exploration of the impacts of imperfect vision and non-representative cross-sections on the ASTM standards measurement analysis techniques. Imaging, “perfect segmentation” (allowed by the synthetic nature of our data), and grain size determination methods are performed on the structures to reveal direct grain-correspondence between the imaged 2D grain cross-sections and 3D “ground truth” information about each grain. Although resource-intensive methods such as atom probe tomography and serial sectioning^17^ can acquire data about the microstructure’s 3D characteristics, synthetic grain structures can achieve this more efficiently and cheaply, providing unparalleled statistics with options to integrate and study other data acquisition artifacts. Additionally, the synthetic grain structures permit the direct comparison of methods that may otherwise be impossible to use on the same volume of a sample.

**Fig. 2:**
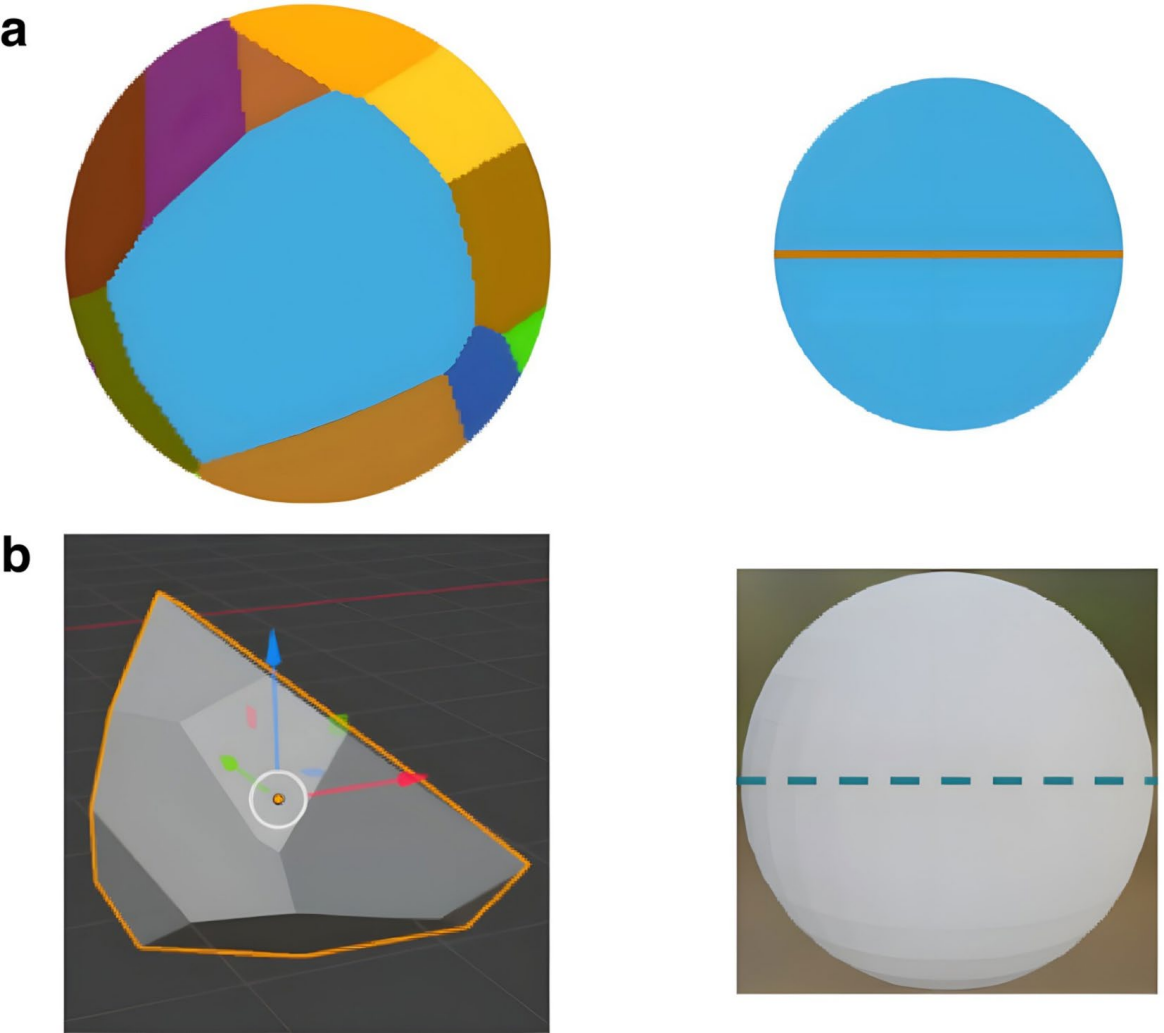
Schematics of the equivalent 2D and 3D diameters used for comparisons between dimensions. (**a**) A cross-section projection of a grain on the left with a circle of equivalent area on the right. The diameter of this equivalent area circle is the 2D diameter. (**b**) Similarly, an individual 3D grain is shown on the left with an equivalent volume sphere on the right. The diameter of this equivalent volume sphere is the 3D diameter.

Two types of 2D cross-section, flat polish (Fig. 1a) and intergranular fractograph (Fig. 1b) are generated and analyzed to establish one-to-one correspondences between 1D grain parameters and 3D measurement data. Grain sizes from the projected cross-sections and the synthetic grain volume itself are compared by using equivalent “2D” and “3D” diameters, as illustrated in Fig. 2. The former is the diameter of the circle with an equivalent area to the cross-section projection of a grain. The latter is the diameter of the sphere with an equivalent volume to a 3D grain. Thus, both the grain areas and volumes can be measured in a manner which allows for a meaningful one-to-one correspondence between 2D and 3D grain measurements. The 3D diameter distribution is less skewed and more symmetric than the volume distribution due to the change of dimensionality, shown for $\alpha$-iron, Al-2%Sn, and Ti-21 S $\beta$ experimentally^14,18–20^.

## Results

### Grain size distributions

Grain size distributions are collected from a synthetic grain structure by taking a fractograph cross-section, flat polish cross-section, and the 3D grain structure itself and calculating all the equivalent diameters. The probability density distributions of the equivalent diameters can then be fitted and plotted.

**Fig. 3:**
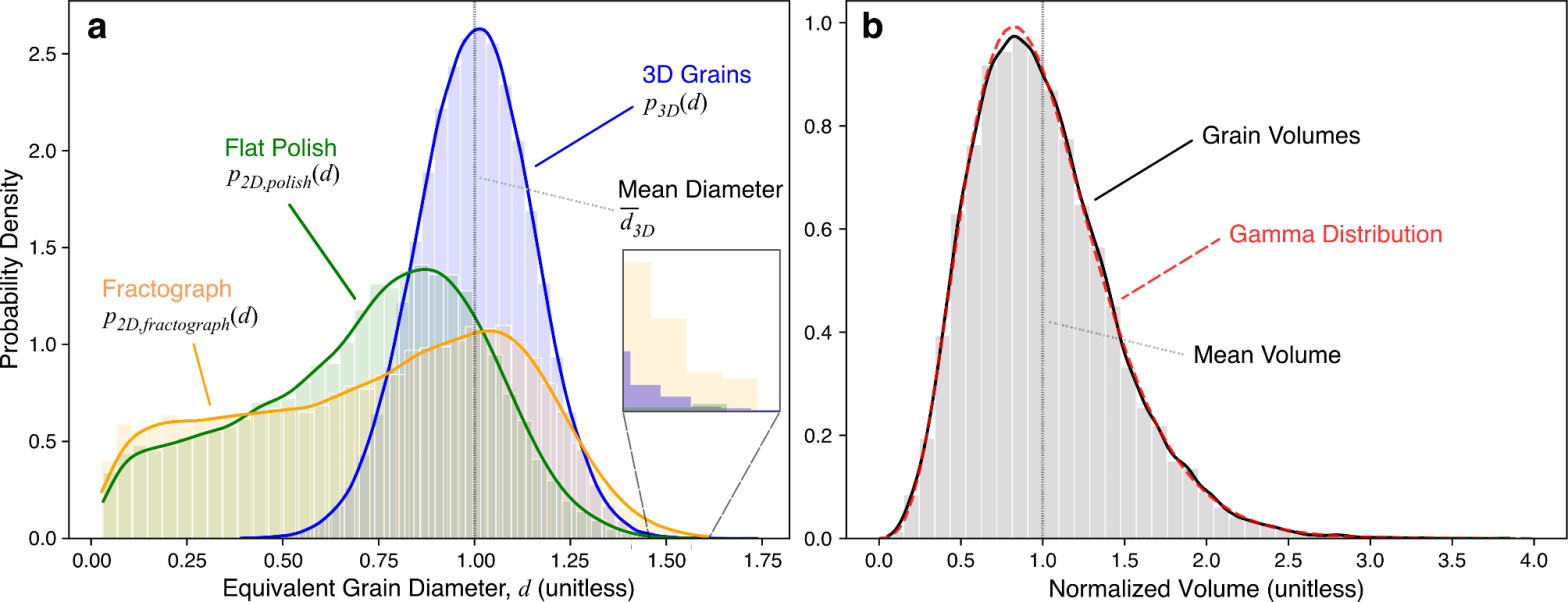
(**a**) Grain size distributions for a synthetic 3D grain structure, polished surface, and fractured surface. The 3D grain probability densities were based on 89,787 3D grains, while the fractograph and flat polish distributions were based on 12,534 and 10,461 2D grains, respectively. (**b**) The normalized volume distribution of the same 3D grains, fit by a gamma distribution ($\alpha=5.93$).

Figure 3a shows the probability density plot of the 3D equivalent diameters alongside the distributions for flat polish and fractograph cross-sections. The distributions presented include measurements aggregated across several synthetic grain structures. The data from each synthetic grain structure is normalized to its own mean 3D diameter such that the combined average is 1. Figure 3b contains the density plot of the corresponding normalized grain volumes, which has a characteristic right skewed gamma distribution^15^.

The 3D diameter distribution, which would typically be unobtainable by cross-section analyses of a real sample, is symmetric. This symmetry results from the Voronoi construction, but is seen in real samples, typically with a larger spread^14^. In contrast, both the flat polish and fractography distributions have a significant left skew. As will be demonstrated, this skew emerges from cross-sections that underestimate true 3D grain size and can unpredictably influence standard ASTM measurement techniques. The mode of the fractograph distribution corresponds closely with the mode of the 3D diameter distribution, while the flat polish mode is smaller. Additionally, the upper bound of the fractograph distribution’s domain is close to the upper bound of the 3D diameter distribution.

The existence of 2D diameters that approach 0 can be explained by small, barely exposed grains that are mostly “buried” by the surrounding grains in the projection. These small 2D diameters may not be visible in practice due to resolution limitations and boundary labels, but uncertainty surrounding the size of this effect could contribute to “imperfect vision” of grain cross-sections in practice.

**Fig. 4:**
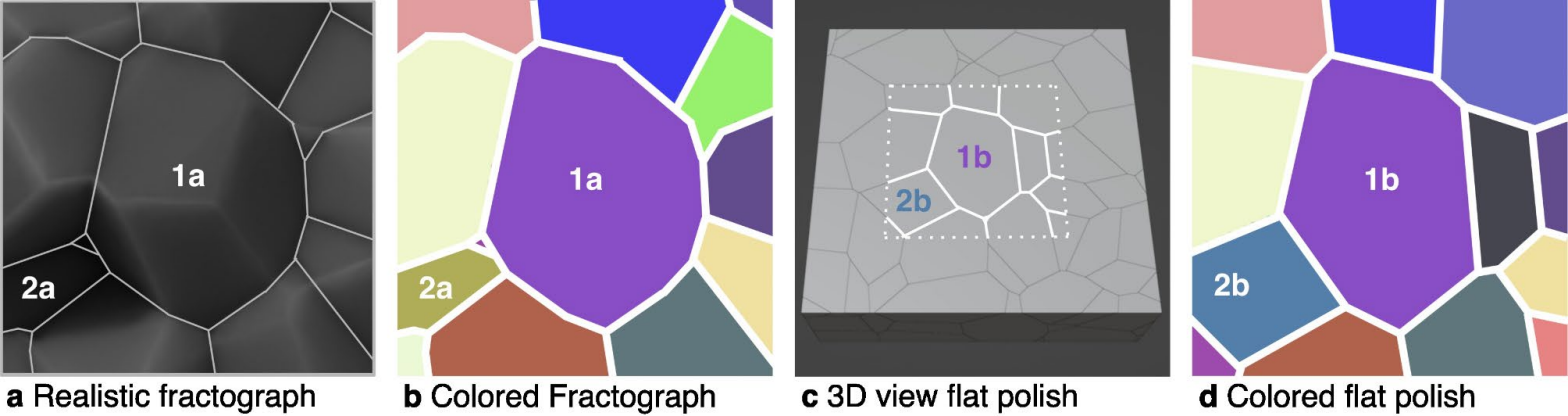
The same surface subsection imaged in four different ways. (**a**) A realistic fractograph rendering of the surface. (**b**) The colored 2D projections of the fractograph surface. (**c**) A flat polish rendering of the same subsection. (**d**) The 2D projections of these grains for the flat polish surface.

Figure 4 shows a set of complementary fractograph and flat polish samples. When creating a flat polish surface on a given plane of material, there are often certain grain projections present that would have been removed had there instead been a brittle fracture along the same plane. In the complementary fractograph, the absence of these grain projections results in the grains immediately underneath being unobscured, enlarging their projection areas.

Figure 4b and d show the change in grain projection areas for this region between the fractograph and flat polish samples. Grain 1a is larger than grain 1b, and the absence of certain grains that were obscuring grain 1a can be seen around the area of grain 1b. This difference in the two samples illustrates how the fractograph distribution realistically has a wider domain and larger mode. Grains 2a and 2b are two different grains; they represent the result of an opposite effect. In this case, that flat polish grain projection 2b (removed in the fractograph, revealing the grains underneath) has a larger area than the revealed grain projection 2a in the fractograph. However, grain 2b can also be seen to be obscuring two grains to either side, which are larger in the fractograph cross-section.

The fractograph distribution in Fig. 3a contains measurements of larger grain projections that are now revealed (e.g. grain 1a in Fig. 4a) due to being unobscured by the removal of surrounding, often smaller (in projected cross-section), grains. This unobscuring implies a better match between the fractograph grain diameter distribution and the 3D grain size distribution among these larger diameters. Correspondingly, the fractograph distribution to the right of its mode aligns more closely with the 3D grain size distribution than its flat polish counterpart.

**Fig. 5:**
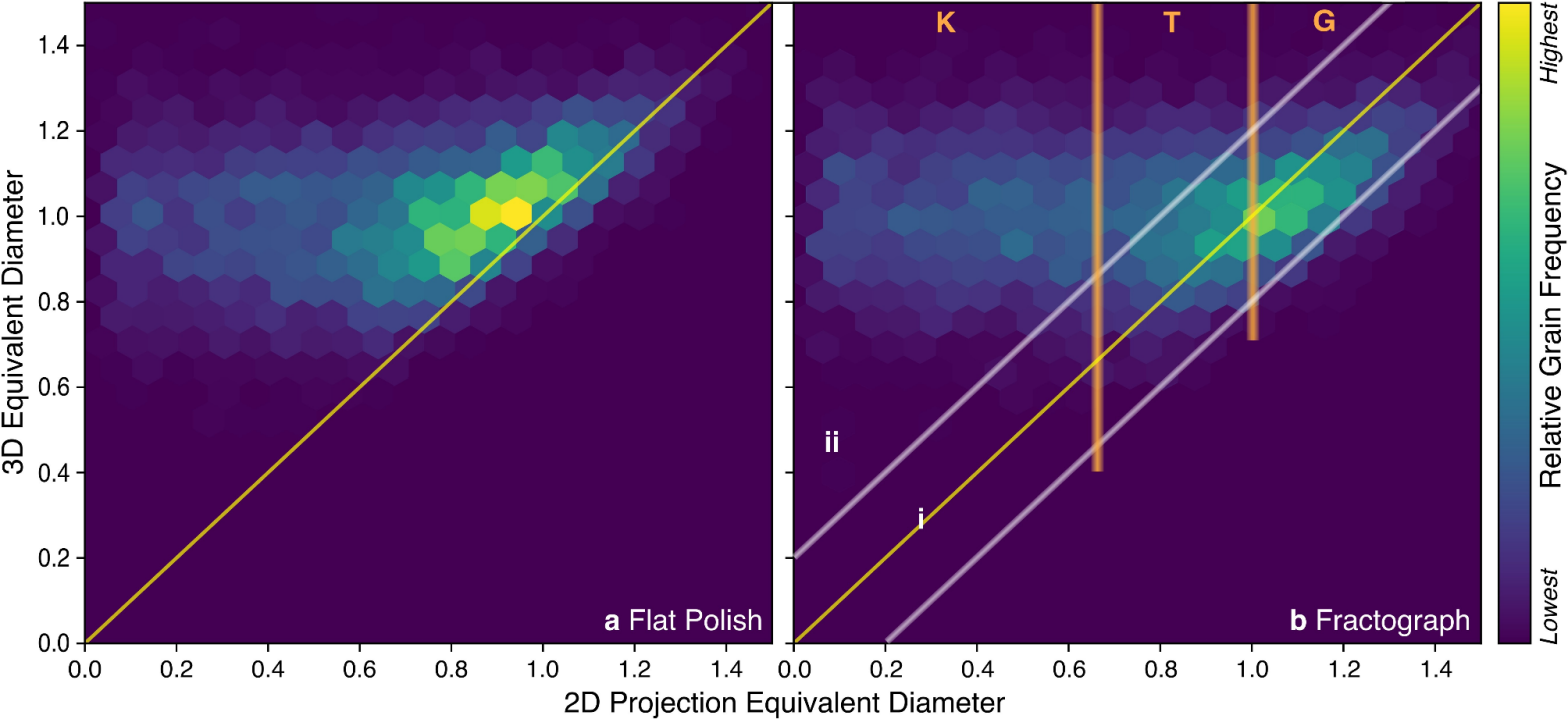
Joint frequency density plots, on the same color scale, derived from one-to-one grain correspondence relating 2D and 3D equivalent diameters for individual grains. The diagonal yellow solid line indicates equivalent 2D and 3D diameters. Grains to the left of the equivalence line are underestimated by their 2D diameter, while those to the right are overestimated. For individual grains in both fractograph and polished cross-sections, measurements can range from large underestimation to slight overestimation of the actual 3D grain sizes. (**a**) Flat polish. (**b**) Fractograph, where regions **i** and **ii** contain grains near and far from the equivalence line, respectively. Regions **K**, **T**, and **G** are used for discussion.

Within each bin from the 3D diameter distribution in Fig. 3, the correspondence of each grain to its projected 2D diameter value is known. The bin’s 2D diameter distribution can be heat-plotted along a horizontal slice which, when combined with the grain data from other bins, yields a “heat map” (Fig. 5). Summing all of the horizontal slices of the heat plot in Fig. 5 results in Fig. 3a. Thus, Fig. 5a and b demonstrate the discrepancy between true 3D grain size and the apparent 2D grain size from 2D projection methods for flat polish and fractograph surfaces, respectively.

As indicated by Fig. 5, the full picture is complex. Individual 2D grain projections can underestimate, be equivalent to, or overestimate the true 3D volume regardless of their relative size in the probability density function or the imaging method. Crucially, one can observe that the “hottest” (highest frequency) area in the fractograph heat map aligns better with the equivalence line compared to the flat polish heatmap. The fractograph distribution centers closely on the actual 3D volume across all sizes across the equivalence line. The demonstrated correspondence between the fractograph 2D diameter distribution and the 3D diameter distribution suggests a measurement procedure to approximate the 3D grain size distribution from 2D fractograph cross-sections.

### Procedure to approximate 3D grain size

The following procedure assumes equiaxed grain structure and an approximately symmetric distribution of 3D grain sizes. We define the distribution of equivalent diameters from a grain cross-section to be ${p}_{2D}\left(d\right)$ and the distribution of equivalent diameters for the 3D structure to be ${p}_{3D}\left(d\right)$. The Eq. 

1 $${\stackrel{-}{d}}_{3D}=k\underset{d}{\text{max}}{p}_{2D}\left(d\right)$$

states that the mean 3D equivalent diameter, ${\stackrel{-}{d}}_{3D}$, is equal to the mode of ${p}_{2D}\left(d\right)$ multiplied by a constant factor $k$.

**Fig. 6:**
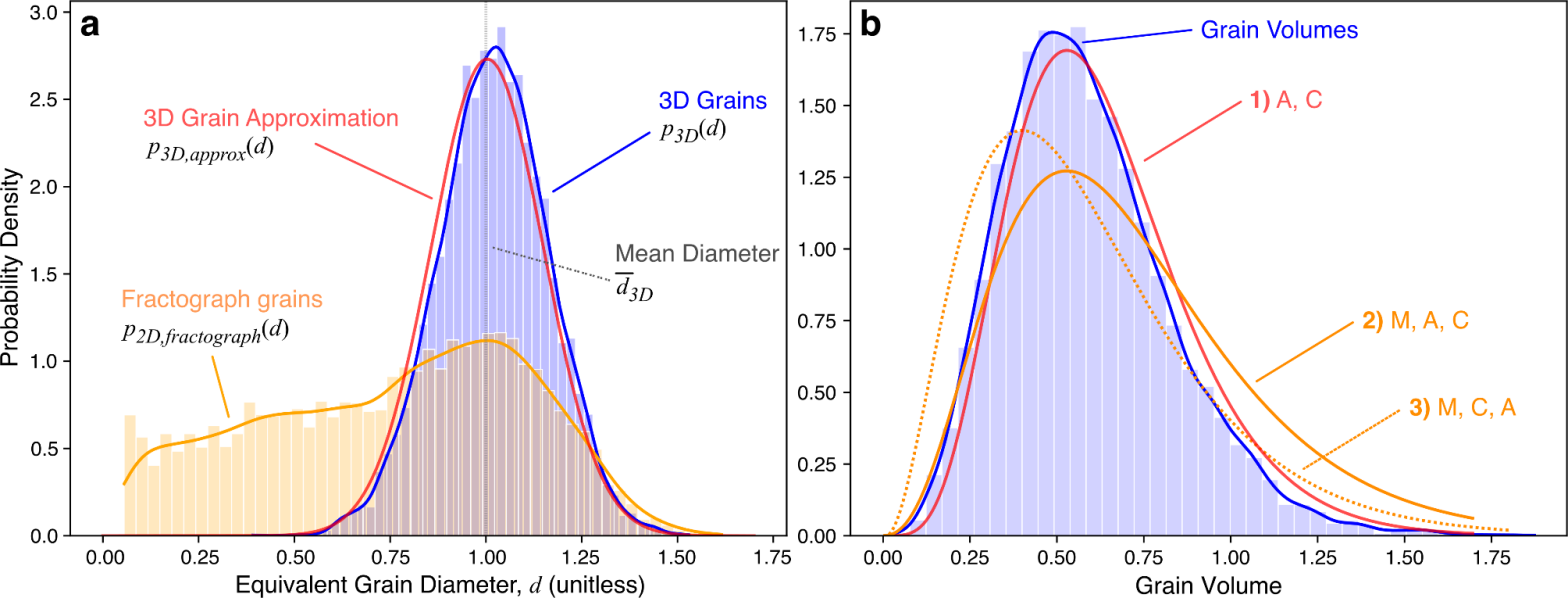
(**a)** Approximated and actual 3D grain size distributions alongside the 2D fractograph distribution. The bins of the fractograph grains represent the sample ${D}_{2D}$. The approximated 3D grain size distribution of a synthetic data sample was derived from its 2D fractograph distribution of equivalent diameters using 15,464 grains and kernel density estimation. (**b)** Three approximations of the grain volume distribution, varying the order of approximation (A), conversion to 3D volume (C), and mirroring of the grain distribution across the mode (M).

The constant factor is determined based on the type of cross-section, flat polish or fractograph, and by the shape of the grains in the structure. For a flat polish surface, as seen in Fig. 3a, $k<1$. For a fractograph surface, since grain cross-sections are less obscured, $k\approx1$. Thus,

2 $${\stackrel{-}{d}}_{3D}\approx\underset{d}{\text{max}}{p}_{2D,fractograph}\left(d\right)$$

holds for a fractograph cross-section. This approximation of the mean 3D diameter, ${\stackrel{-}{d}}_{3D}$, is shown as the peak of ${p}_{3D,approx}\left(d\right)$in Fig. 6a.

The data required to characterize ${p}_{2D}\left(d\right)$ to approximate ${\stackrel{-}{d}}_{3D}\:$is more detailed than what is traditionally collected using the ASTM standard methods. We are assuming that the 2D equivalent grain sizes of the individual grains in the image can be calculated with the assistance of image analysis or advanced segmentation models. We will demonstrate that, while requiring more data to lower the variance of the measurement, this new method could prove more robust to differing imaging conditions that obscure small grains by eliminating reliance on smaller grain projections.

### Approximating the equivalent diameter distribution

For estimating the spread of the 3D grain size distribution in Fig. 6a, we assume the largest grain size in the 2D distribution sample ${D}_{2D}$ corresponds closely with the largest 3D grain in the distribution. This largest grain size value can be used to approximate the spread, or standard deviation, of the grain size distribution. As demonstrated in Fig. 6a, given that the 3D distribution is assumed normal, the spread is approximated according to the following equation:

3 $${s}_{3D}\approx\frac{{d}_{2D\:max}-{\stackrel{-}{d}}_{3D}}{\text{log}n}$$

Here, $s$ is the estimated spread of the 3D distribution, ${d}_{2D\:max}$ is the largest observed equivalent diameter of the 2D distribution, ${\stackrel{-}{d}}_{3D}$ is the result from Eq. 1, and $\text{log}n$ is the order of magnitude for the number of grain cross-sections used.

This approximation is based on that, in a normal distribution, the probability of sampling a larger value gets exponentially smaller as the value gets further from the mean. For a sample of $n=1000\:$grains, $\text{log}\left(n\right)=3$ implies that the largest measured diameter (${d}_{2D\:max}$) is expected to be 3 standard deviations from the mean (${\stackrel{-}{d}}_{3D}$). This roughly corresponds to the 0.27% chance that a random 3D grain diameter is ${3s}_{3D}$ greater than ${\stackrel{-}{d}}_{3D}$. Thus, with $n=1000$, one might expect 2–3 grains to be 3 standard deviations away, so it is reasonably safe to use ${d}_{2D\:max}$. This expectation becomes less reasonable as sample size decreases, in which case mirroring ${p}_{2D,\:fractograph}\left(d\right)$ about its mode using Eq. 5.2 and taking the standard deviation (making use of more grain projection samples) is another option.

Combining the results from Eqs. 2 and 3, we can derive the approximation of the probability density function for the 3D equivalent diameter distribution, represented by the random variable ${D}_{3D,\:approx}$:

4 $$P({D}_{3D,\:approx}=d)={p}_{3D,approx}\left(d\right)\:\sim\:N\left({\stackrel{-}{d}}_{3D},\frac{{d}_{2D\:max}-{\stackrel{-}{d}}_{3D}}{\text{log}n}\right)$$

where $N$ is the normal distribution.

### Approximating grain volume distributions

Methods to approximate the grain volume distribution from a lower dimensional cross-section can also be derived based on the previous results. These methods consist of three operations. Approximating (A) is applying a curve-fit to the present data. Conversion (C) turns 3D equivalent diameters into a volume measurement. Mirroring (M) takes the grain diameters to the right of the peak in the sample ${D}_{2D}$ and mirrors them across the mode value ${\stackrel{-}{d}}_{3D}$, using Eq. 5.2. This operation, good for lower grain sample sizes, relies on more points and constructs an approximate, symmetric histogram.

5.1 $${D}_{2D,half}=\left\{{d}_{i}\in\:{D}_{2D}|{d}_{i}\ge\:{\stackrel{-}{d}}_{3D}\right\}\:$$

5.2 $${D}_{2D,\:mirrored}={D}_{2D,half}\cup\:\left\{2{\stackrel{-}{d}}_{3D}-{d}_{i}|{d}_{i}\in\:{D}_{2D,half}\right\}$$

The first method of Fig. 6b in red (A, C) approximates the blue (3D grain) curve as in Fig. 6a and Eq. 4 before applying the following conversion into volume:

6 $$V=\:\frac{\pi\:{{D}_{3D,\:approx}}^{3}}{6}$$

The quality of this approach relies on the approximation of the equivalent diameter distribution. The second method (M, A, C) constructs the modified sample ${D}_{2D,\:mirrored}$ by Eq. 5.2 and fits a normal (symmetric) distribution, before transforming the distribution to grain volume using Eq. 6. Finally, the third method (M, C, A) applies the mirroring operation, converts each individual grain in ${D}_{2D,\:mirrored}$ to a grain volume, and then fits the gamma distribution to the grain volumes using statistical software^21^.

Here, the first (A, C) and second (M, A, C) methods line up the approximation’s peak well since the volume conversion takes place after the approximation. The third method (M, C, A) suffers from a “stretch” effect during conversion to volume. This method misaligns the peak of the volume distribution but characterizes the spread better than the second method. When approximating the up-dimensional distribution, this “stretch” effect, decided by the sequence of operations, should be considered.

### Robustness of the approximation method to imperfect vision

Ideally, image analysis would have “perfect vision” free of artifacts and resolution limitation such that grain boundaries can be accurately delineated. However, in practice, there may be a limit to the number of grains that can be imaged with sufficient resolution for grain boundary identification^22^. In this section, we model this imperfect vision and segmentation by assuming that they disproportionately affect smaller grain projections in the image.

**Fig. 7:**
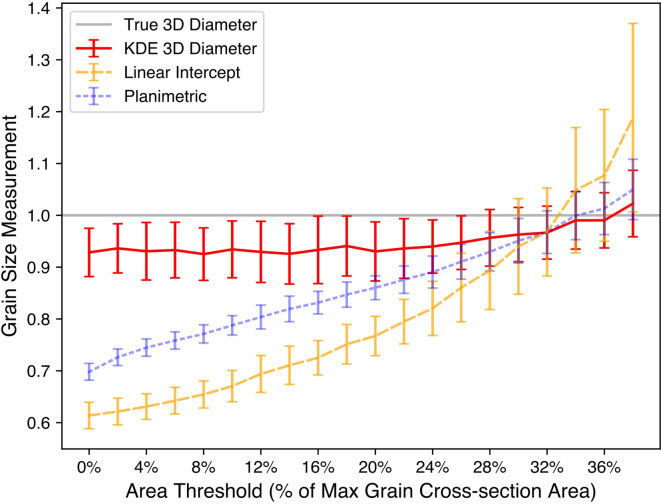
3D grain size approximation, Heyn linear intercept, and Saltykov planimetric methods plotted across levels of imperfect imaging conditions. Imperfect imaging conditions are modeled by ignoring grains below a certain area projection threshold while performing the procedures as they otherwise would be performed. The area threshold is taken as a percentage of the max grain cross-section area, which corresponds to a grain roughly 1.6x the average 3D grain size. The 3D approximation uses the mode of the fractograph distribution based on kernel density estimation (KDE). Each set of measurements along the x-axis represents 250 random samplings of cross-sections including roughly 180 grains each. The error bars show standard deviations. For the 3D approximation method, the ignored grains are not included in the 2D projection distribution prior to performing it. For both ASTM standard methods, the small grain area is effectively distributed amongst all the remaining grains due to not counting them (see Methods, Implementing Standard Grain Size Analysis Techniques, Eqs. 7–10).

In our model, we start with a baseline perfect vision by considering every grain in the cross-section and then systematically increase the degree of imperfect vision by raising a minimum grain area threshold for each grain to be considered. This is also analogous to having different microscope settings where the same area of interest is imaged with a lower pixel count or a less well aligned electron beam. Across varying degrees of this imperfect vision, we ran the 3D grain size approximation, Heyn linear intercept, and Saltykov planimetric methods on numerous samples, seen in Fig. 7.

For a given set of imaging conditions, it is difficult to know precisely what amount of “imperfection” exists. Consider Fig. 7 where, as small grains are disproportionately impacted, the imperfection level has a considerable impact on the conventional standard measurement methods. Both ASTM standard methods are also shown to underestimate ${\stackrel{-}{d}}_{3D}$ due to the heavy left tail of ${p}_{2D}\left(d\right)$; interestingly, they also approach ${\stackrel{-}{d}}_{3D}$ by an uncertain amount related to the degree of imperfect vision. By contrast, the mode approximation method does not rely on small grains and is much more robust across a range of imperfection levels.

This robustness falters once the minimum area threshold gets close to the mean itself, but such a case would be assuming a microscopist who cannot image an average grain in focus and may therefore be duly ignored. Thus, the most practical range shown in Fig. 7 is roughly between 0 and 8%, as these assume well imaged cross-sections and contain minimal effects from the effective distribution of ignored grains’ projections to the remaining grains.

The ${\stackrel{-}{d}}_{3D}$ approximation using KDE has a higher standard deviation than the other methods. However, this is expected since the measurement method relies on the mode, which can be more susceptible to random sampling when less data is used. To counteract this, the precision of the 3D grain diameter approximation can be increased by increasing the number of grains sampled, $N$. Machine learning (ML) models that automatically label fractograph grain boundaries could play a large role in collecting enough data to allow the standard error of the 3D approximation method to be comparable to the standard methods.

## Discussion

### Theoretical basis for the approximation procedure

The 3D grain size approximation method outlined in Eqs. 2, 3 and 4 can be explained by considering the random sampling of cross-sections from a *single*,* equiaxed*,* and convex* grain. Keeping the same orientation, there is a single plane that produces the cross-section with the maximum area, likely close to the middle of the grain. On either side of this maximizing plane, all cross-sectional area projections are smaller.

**Fig. 8:**
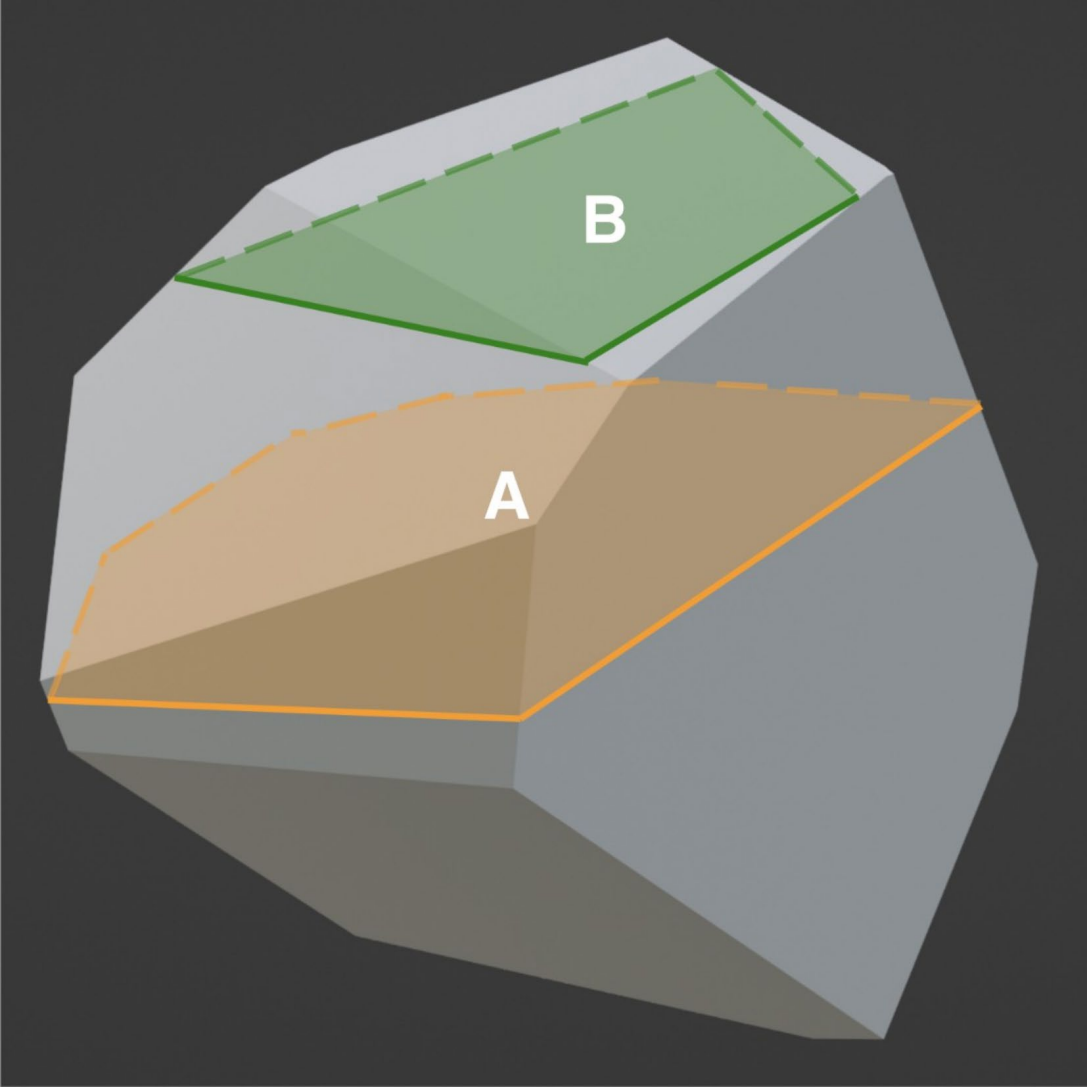
A single convex grain with two labeled cross-sections. Certain cross-sections such as A can produce more representative equivalent diameters compared to underestimating cross-sections such as B for a single given grain. The maximal cross-section implies that underestimations of grains of this size interfere with information provided by smaller grains more so than larger grains.

Thus, while randomly sampling the 2D equivalent diameters of a single, equiaxed grain at a given orientation like in Fig. 8, one would expect a maximum possible value corresponding closely to the 3D equivalent diameter of the grain, illustrated with cross-section *A*. Any other cross-sectional 2D diameters could range from 0 to this maximum 2D equivalent diameter, such as *B*. This is analogous to taking the equivalent diameters of spherical cross-section from a sphere, which range from 0 to the sphere’s diameter at the center. Notably, the maximum 2D diameter of an irregularly shaped (but almost always convex) equiaxed grain could result in a value slightly larger than the equivalent 3D diameter. Drawing 1D lines on the projected areas of imaged 2D grains and plotting the cord length distribution results in a similar underestimation effect but at this lower dimension.

Now, consider an entire distribution of packed equiaxed grains. When a cross-sectional surface is exposed, the total area of all grains *imaged* is the same, regardless of whether a polished or fractured surface is used. However, the distribution and number of projected areas are different in the two cases, and hence the 2D diameter distributions also differ. In both surface exposures, the resulting areas for each grain are typically underestimations of their actual size, but more so for a flat polish cut, as seen in Fig. 3a. A less obscured grain from a fractograph, as shown in Fig. 4, becomes akin to viewing an individual grain from above. Thus, the resulting 2D equivalent diameter becomes closer to the upper limit: the grain’s actual 3D equivalent diameter. This intuition corresponds with the higher frequency densities for the fractograph heatmap near the equivalence line in Fig. 5b.

The result is that the aggregate distribution of the fractograph corresponds closely with the shape of the true ${p}_{3D}\left(d\right)$ to the right of its peak (i.e. grains larger than the mode). As visualized in Fig. 5b, the value of ${p}_{2D,\:\:fractograph}\left(d\right)$ represents the projections of individual 3D grains that either are roughly equal to the equivalence line and therefore a good estimate of the true 3D diameter (region **i**), or are not (region **ii**). These projections can be divided into distinct regions. Within region **G**, where ${\stackrel{-}{d}}_{3D}\le\:d\le\:{d}_{3D,max}$, the case where 2D equivalent diameters are a good 3D estimate dominates and should be used. In region **K**, where ${d<d}_{3D,min}$, all grain projections underestimate the actual 3D grain and should not be used. Region **T**, where ${d}_{3D,min}\le\:d\le\:{\stackrel{-}{d}}_{3D}$, marks the transition between regions **G** and **K** where there is an increased prevalence of 2D diameters that underestimate the true 3D grain size; its use is not recommended. Within ${p}_{2D,\:\:fractograph}\left(d\right)$, the 2D equivalent diameters in regions **K** and **T** lose correspondence with ${p}_{3D}\left(d\right)$ and instead constitute the significant left tail of the distribution.

Therefore, the “peak,” or statistical mode, of the fractograph distribution corresponds closely to the actual mean grain size of the 3D grain size distribution, explaining the approximation of ${\stackrel{-}{d}}_{3D}$ in Eq. 1. Likewise, the maximum cross-sectional area sampled in ${p}_{2D,\:\:fractograph}\left(d\right)$ corresponds closely with the maximum grain size in the distribution of 3D grains, justifying the assumption used in Eq. 3.

In essence, an “up-dimensional” approximation of 3D grain size from a 2D cross-section is possible because: (1) *equiaxed grains are mostly convex and roughly symmetric*, (2) *each sampled grain cross-section has an upper limit set by its 3D grain size*, and (3) *there exists a mode value in the underlying distribution*${p}_{3D}\left(d\right)$. These reasons are agnostic to the Voronoi tessellation method or grain model used, although the spread of ${p}_{3D}\left(d\right)$ can reduce the factor $k$ in Eq. 1. Together, these underpinnings suggest a stable approximation of the average 3D grain size, ${\stackrel{-}{d}}_{3D}$. Upon considering rough symmetry in 3D equivalent diameter distributions^14^, the 2D diameters ${d}_{2D}>{\stackrel{-}{d}}_{3D}$ such as ${d}_{2D,max}$ can be used to approximate ${p}_{3D}\left(d\right)$ itself, which can be converted to a gamma distribution of grain volume.

### Uncertain effects of 2D artifacts

Various factors influence the relationship between the standard grain size measurements based on metallography images and the “ground truth” 3D size of grains. These factors can also influence the relationships and comparability of methodologies across experimental conditions. Most prominently, small grains are likely to be missed due to factors such as magnification, shadowing, variations in depth, resolution, and image noise.

The resolution will determine the threshold at which small grains will be ignored, or the “imperfection level” from Fig. 7. Variations in depth in fractograph images can also result in “shadows” that obscure smaller, deeper grains. Magnification controls to what extent these factors affect measurements. At one extreme, the magnification may be too low to see any grains clearly. At the other extreme, smaller grains may become revealed, but the number of larger grains in the image is also reduced, skewing measurements in the opposite direction.

The amount of bias caused by this incomplete information will remain difficult to determine in practice. However, the mode of the 2D grain size distribution stays intact regardless of these factors, provided only relatively smaller grains are affected such that determination of the mode of the distribution is not impacted. Thus, the proposed procedure has the advantages of being more “forgiving” on magnification requirements and of yielding a more consistent measurement across experimental conditions, as shown by Fig. 7, as the grain size distribution estimation does not depend strongly on the smaller grains.

### Use in additional cases

As mentioned earlier in Eq. 1, the projected areas can overestimate the true equivalent 3D diameter by a certain factor $k$ dependent on its shape. As grains become more spherical, $k\:$approaches 1, but they also lose the ability to fully pack Euclidian space. The factor $k$ can vary based on the type of surface and general shape of the grains along a given surface plane. For an equiaxed, unimodal distribution of grain sizes on a fractography surface as studied in this work, $k\approx\:1$.

The results have implications for flat polish surface as well. The 3D approximation method, when applied to a fractograph, results in a 1D grain size parameter that is robust to imaging noise (Fig. 7) and approximates ${\stackrel{-}{d}}_{3D}$ (Fig. 6a). When the method is applied to a flat polish distribution, the resulting grain size parameter would have a smaller value of $k$ and require an offset adjustment to correct for the underestimation of the true 3D grain size (see Fig. 5a).

With regard to more complex grain structures that are multi-phase, bimodal, or not equiaxed, one could subdivide the analysis of grains based on the identified phase and direction of the stereological plane, allowing for an analysis to be performed on the separate phases and directions to yield a full picture of the structure. This is similar to the methods suggested by the ASTM standards when applying the traditional standard methods to more complex grain microstructures.

## Conclusion

The synthetic generation of grain structures using a Voronoi construction permits a massive collection of data with more complete, 3D information than practically attainable. Such data facilitate an assessment and analysis of commonly used ASTM procedures, shedding light on their discrepancy with the true 3D distribution parameters, especially due to over-consideration of smaller grain area projections. Additionally, imperfect vision and segmentation stemming from resolution and imaging methodology can introduce an unpredictable error affecting these stereological techniques. This is not of practical importance when grain size tolerances for desired properties are large, but these biases could affect comparisons of different samples that require more refined analysis. Using synthetic data, it is possible to model the “ground truth” against which to compare and quantify the differences due to image acquisition and analysis artifacts.

By comparing the distribution of 2D equivalent diameters in a cross-section with the 3D equivalent diameters of the entire structure, we can show that the distribution of fractograph diameters corresponds closely to that of the 3D diameters for grain sizes greater than the mode. By contrast, smaller 2D grain diameters can just as easily reflect either a similar 3D grain diameter or a cross-section that underestimates a much larger grain. Since there is a decisive upper limit for the cross-sectional area that can be extracted from the lower dimensional 2D projection of a grain, this correspondence between the 2D and 3D grain diameter distributions above the mode value allowed the authors of this work to propose a new grain size and volume distribution approximation procedure that holds particularly well for fractographs due to the un-obscuring of grain areas that occurs during a fracture.

Within this procedure, the statistical mode of the equivalent 2D diameter is used. Provided that enough grains are sampled to reveal a mode value and a maximizing cross-section of one of the largest grains, the lack of dependence on the left tail (smaller grain projections) of the 2D grain distribution makes the method robust to the uncertain bias resulting from imperfect and varying imaging conditions. Thus, the spread of the distribution can be approximated using the estimated ${\stackrel{-}{d}}_{3D}$ and the maximum 2D grain diameter that is sampled, ${d}_{2D,max}$.

This procedure for approximating grain size distributions does not require changes to existing image acquisition and segmentation techniques, but it does require a more substantial analysis compared to the planimetric and line intercept methods. In exchange, it delivers an “up-dimensional” approximation previously not considered. This is particularly relevant in situations where detailed comparisons of material properties beyond what can be obtained from ASTM standards is important, and when grain size interpretation consistency across experiments and research groups is critical.

In this work, the cases of equiaxed grains for fractography and flat polish surfaces were considered. For future work, the Voronoi construction can be adjusted to model other types of grains or specific materials by using different tessellation techniques (e.g. Hardcore, Laguerre) which provide a 3D ground truth reference. Another potent spinoff application using the realistic synthetic grain structure data is for training artificial intelligence / machine learning (AI-ML) models to automatically perform instance segmentation (grain labeling) on fractograph or polished cross-sections. The fact that any images thus generated come with the ground truth can dramatically reduce – if not eliminate – human-induced or acquisition-related labeling bias and imperfections. These structures can be generated and fine-tuned to specific cases in huge numbers to provide training data, opening a potentially transformative path to expediting AI-ML development in fundamental materials science.

## Methods

### Generating synthetic grain structures

We performed a Poisson Voronoi tessellation using the “cell fracture” add-on in the computer graphics software application Blender^23^. Starting with a rectangular prism, this add-on takes a random distribution of points and subdivides the object into smaller cells via a Voronoi tessellation^24^. This process yields a random, simulated grain structure (Fig. 1a) where the 3D grain diameters are symmetrically distributed and the grains are equiaxed.

Each grain is colorized with a unique color value and zero shading (Fig. 1c) such that every grain can be matched with its projection in a 2D cross-section. Random colorization yields a convenient unique identifier that ensures each grain can be matched to a cross-section for one-to-one comparisons. Prior to the taking of simulated cross-sections, the 3D information of each grain is saved and linked to its unique identifier.

### Synthetic cross-sections

In this study, two types of 2D cross-sections (“intergranular fracture surface” and “polished surface”) are generated and analyzed for one-to-one grain parameter comparisons.

*Fractograph* cross-sections are generated by iterating over the grains in the synthetic structure and deleting grains that fall outside a specified range or function. The function applied can yield both a planar fracture as well as other practical fracture landscapes such as image tilts and larger variations in depth. Planar fracture cross-sections are relevant to brittle materials that fracture in an intergranular mode, such as the transverse cross sectional fracture of Nb_3_Sn within a uniform filament^25^, whereas an undulating fracture landscape can simulate the longitudinal fractographs of Nb_3_Sn filaments^26^. In this paper, we focused on planar fracture cross sections.

*Polished surface* cross-sections are simulated by using a flat bisection cut across the grain structure along a plane. The typical physical preparation of a flat polished surface involves grinding and fine polishing to expose an artifact-free microstructure for examination and etching if necessary to reveal the grains for microscopy. This serves to remove uneven heights and a damage layer at the surface of the sample. Creating a polished surface is the most common metallographic sample preparation; it is correspondingly the assumed cross-section type for most standard methods of measuring grain size.

Data about the 2D cross-sections such as area, diameter, eccentricity, and perimeter are extracted using the scikit-image^27^ and scipy^21^ libraries. This data then needs to be scaled to match the scale of the grain structure in 3D space. This scaling ratio can be determined for any given virtual magnification by rendering a simple object such as a rectangle of known length (in the arbitrary length units in the 3D environment). The pixel length of the object can then be measured, and a pixels-per-length unit ratio can be determined. The ratio is dependent on the camera’s distance from the surface, resolution, and aspect ratio of the render within the simulated environment.

### One-to-one grain matching

The colored cross-section is processed to extract information about each individual grain projection, including its color, area, perimeter, and bounding box. Knowledge of the bounding box allows for any future processing to ignore grains that are cut off on the edge of the frame; something which must be done to avoid underestimating the areas of any 2D grain projections. The color acts as a unique identifier so that information about each grain’s 2D projected cross-section can be matched with its 3D counterpart in the synthetic model. The result is a one-to-one correspondence of individual grains in the cross-section with data about their shape and size in the full 3D structure.

To facilitate a properly scaled comparison, the one-to-one correspondence is used to calculate an equivalent 2D and 3D diameter for each individual grain shown in the cross-section. These refer to the circular and spherical equivalent diameters, as demonstrated in Fig. 2. For example, a grain with a cross-sectional surface area of π has an equivalent circular diameter of 2 (referred to as the 2D diameter). Similarly, a grain with an actual volume of $\frac{4}{3}\pi\:$ would have an equivalent spherical diameter of 2 (referred to as the 3D diameter). These equivalent diameters are calculated from the areas and volumes when combining the data.

### Implementing standard grain size analysis techniques

Two grain size analysis techniques from the ASTM standards^3^ were implemented: the Heyn line-intercept procedure and the Saltykov planimetric method. These are methods that are used with cross-section images of grains to extract information regarding the average grain size in the form of 1D grain size parameters.

The line intercept is a 1D technique that yields an average diameter by, in one form, drawing many lines across a cross section and counting the number of grains intercepted^28^. The lines should be as long as possible and vary in rotation; the end grains that the line does not pass fully through are counted as half a grain. Equation 7 below yields the average lineal intercept $\overline{l}$ by adding the lengths, ${L}_{i}$, of all $n$ drawn lines and dividing them by the number of intercepted grains ${N}_{I}$. Equation 8 yields ${N}_{l}$, the average number of intercepts per unit length of test line.

7 $$\stackrel{-}{l}=\frac{\sum\:_{i=1}^{n}{L}_{i}}{{N}_{I}}$$

8 $${N}_{l}=\frac{1}{\stackrel{-}{l}}$$

The linear intercept method for determining grain size is implemented using scikit-image^27^. The implementation takes a grain image where each grain has a unique color identifier, as shown in structure Fig. 1c, and draws a specified number of lines, keeping track of total length and the number of unique grains encountered. Obtuse grains that partially wrap another grain, although not occurring in the structures used, would not be double counted. The function to determine the mean linear intercept can run thousands of times per grain projection, collecting an accurate and consistent value. Finally, the result is scaled back from pixels to blender units for comparison to 3D values.

The planimetric procedure consists of drawing a known area over the cross-section and counting the number of grains it inscribes, where grains intercepted partially at the boundary are counted as a half grain^29^. Rather than using a known circular area, Saltykov proposed using a rectangular area in order to reduce the bias in the counting procedure^30^. Thus, Eq. 9 yields the number of encapsulated grains, ${N}_{A}$, for a rectangular area. ${N}_{intercepted}$ ignores the corner grains, which are instead counted as $\frac{1}{4}$ a grain each, resulting in adding $1$. Equation 10 yields the average area of each grain, $\stackrel{-}{A}$, given ${N}_{A}$ and the known area $A$. A 1D diameter parameter is obtained by $d=\sqrt{\stackrel{-}{A}}$.

9 $${N}_{A}={N}_{Inside}+0.5{N}_{intercepted}+1$$

10 $$\stackrel{-}{A}=\frac{A}{{N}_{A}}$$

The Saltykov planimetric method is implemented in Python with scikit-image to draw a rectangle on the image and count the number of grain projections contained within it based on Eq. 9.

### Calculating the mode 2D diameter

#### Binning method

The sampled 2D equivalent diameters, ${D}_{2D}$, from the grain cross-sections can be binned into a histogram. For a fractograph cross-section, the middle of the peak binning region can then be taken as an estimate of ${\stackrel{-}{d}}_{3D}$. Using a binning method by itself is an ideal strategy when the sample size of cross-section grains is high enough to reveal a clear peak in the distribution.

However, the choice of binning scheme can unpredictably affect results, especially for small sample sizes. It is important to understand the effects that one’s binning method will have on results: bins that are too large will offset trends by hiding important details (“low pass”), while bins that are too small will make measurements far more susceptible to random noise (“high pass”). Additionally, there is a relationship between the domain of ${p}_{2D}\left(d\right)$ and the number of bins that can offset results. To alleviate such issues, one possible method is to run multiple analyses with different bin sizes and average each of their ${\stackrel{-}{d}}_{3D}\:$approximations from Eq. 1.

### Median filter

A median filter can be applied to the sample ${D}_{2D}$ before taking the mode of the binned equivalent diameters in scenarios where the sample size is not high enough to decisively determine a peak. The result is an adjusted binned distribution where the value in each new bin is set equal to the median of the region within a certain window size of each old bin. This operation effectively smooths the distribution and can help prevent the selection of an underestimating approximated grain size which can arise especially at low sample sizes due to the heavy left tail as shown in ${p}_{2D}\left(d\right)$ in Figs. 3a and 6.

### Kernel density estimation (KDE)

Another method for finding a peak value is by using a continuous approximation of ${p}_{2D}\left(d\right)$, such as Scott kernel density estimation^31^ as used in Fig. 7. For this method, an appropriate kernel size needs to be selected so that the density estimation does not over-fit the sampled data. The kernel density estimation built into libraries such as Seaborn^32^ and Matplotlib^33^ may also be used.

Once a smooth approximation of ${p}_{2D}\left(d\right)$ has been obtained, its mode can be taken as the approximation of ${\stackrel{-}{d}}_{3D}$. The KDE method performs best when the sample size is high enough to see either a clear distribution or slight variations about an otherwise observable shape. If there are large spikes in the lower grain sizes of the distribution, other options such as the median filter or collecting more samples may be more ideal.

## Data Availability

The datasets generated during and analyzed during the current study are available from the corresponding author on reasonable request.
